# Water-Cooled Radiofrequency: A Neuroablative or a Neuromodulatory Modality with Broader Applications?

**DOI:** 10.1155/2011/263101

**Published:** 2011-12-18

**Authors:** Khalid Malik, Honorio T. Benzon, David Walega

**Affiliations:** Department of Anesthesiology, Feinberg School of Medicine Northwestern University, Chicago, Il 60611, USA

## Abstract

We report the successful use of water-cooled radiofrequency where more traditional forms of neuroablation—conventional and pulsed radiofrequency—had failed to achieve adequate pain relief. We also discuss the mechanism of neural damage with water cooled radiofrequency and discuss why this technique may have a broader role in the management of a wide array of pain syndromes.

## 1. Introduction

Water-cooled radiofrequency (WC-RF) ablation is a minimally invasive neuroablative technique used in the treatment of various pain syndromes. Even though WC-RF has been used for some time in cardiac electrophysiology [1] and tumor ablation [2], its use in the treatment of pain is recent. The mechanism of pain relief from WC-RF application is analogous to conventional thermal radiofrequency (CT-RF) application—a thermal lesion is created, by applying radiofrequency (RF) energy through an electrode placed in the vicinity of the target neural structure, with the aims of interrupting the afferent nociceptive pathways. In contrast to CT-RF, however, the volume of the tissue heated and the resultant thermal lesion is substantially larger with WC-RF application [1–3]. The larger area of neurodestruction increases the probability of successful denervation of a pain generator with numerous and/or variable afferent nociceptive innervation [3, 4]. With the numerous and variable sources of innervations, the intervertebral disc and sacroiliac joints are currently the exclusive targets for WC-RF. Due to its ability to precisely deliver thermal energy to larger tissue volumes, WC-RF may well be effective when conventional forms of neuroablation are unsuccessful. Based on this assumption we deem that WC-RF may be employed in treating other pain syndromes. Here we report the successful use of WC-RF in two cases when established methods RF applications had been ineffective. 

## 2. Case Report 1

The patient is a 71-year-old retired ophthalmologist with left-sided low-back pain of several months duration. On examination, he exhibited significant tenderness of the left low-back paravertebral area and his pain worsened with low-back extension. After a preliminary diagnosis of left lower lumbar facet arthropathy the patient was treated with a series of lumbar facet joint injections of local anesthetic and steroid—0.5% bupivacaine 1.5 cc with 20 mg of methylprednisolone per joint. The patient reported adequate but temporary pain relief from these injections. Three months after the initial presentation CT-RF of the left L2 through L5 medial branches was performed. The patient reported minimal pain relief from the procedure at 3- and 12-weeks after the procedure. Six months after the initial presentation WC-RF lesioning was performed at the left L2 through L5 medial branches using specialized 18 G WC-RF electrode with 6 mm active tip and the maximum electrode temperature raised to 55°C for 150 seconds. At 3 and 12 week follow-up visits, the patient reported significant pain relief with pain scores reduced to 2-3/10 (NRS). He had resumed his daily activities and he was satisfied with the overall results of his treatment. Our technique of electrode placement for WC-RF was similar to CT-RF of medial branch described in previously published studies [5]. Nevertheless, due to the larger expected lesion size we ensured a “safe distance” from the segmental spinal nerve by adopting the following safety measures: (a) the electrode tip was placed on the transverse process, about 4 mm lateral to its junction with the superior articular process; (b) the parameters used for sensory testing were modified and paresthesia was sought at >0.8 V, between 0.8 V to 1.0 V.

## 3. Case Report 2

The patient is a 52-year-old school teacher with a history of surgery for ruptured intracranial aneurysm. The patient presented almost 2 years after the surgery with persistent pain in the left parietal area at the site of her surgical incision. Examination of the painful area showed healed surgical scar and marked local tenderness and hyperalgesia. The patient reported her pain as severe, rating it 8-9/10 on numeric rating scale (NRS), which precluded her from performing the activities of daily living. A possible explanation of this patient's persistent pain was neuritis and neuroma formation at the site of surgical trauma. A series of local anesthetic and steroid injections—0.5% bupivicaine with 40 mg of methylprednisolone—of the painful area over the next several months provided only temporary relief. Six months after the initial presentation pulsed RF was applied to the painful area in a fan-like manner creating multiple lesions. The patient reported no noteworthy pain relief after the procedure. Almost two years after the initial presentation and unrelenting pain, WC-RF was applied to the painful area. The specialized 18-gauge water-cooled electrode with 6 mm active tip was used and local temperature was raised to 40°C for 150 seconds. At 3- and 12-week followup the patient reported considerable pain relief with pain scores reduced to 1-2/10 (NRS); she had resumed her daily activities and was satisfied with the general outcome of her treatment.

## 4. Discussion

During RF application the electrical energy imparted to the tissues surrounding the electrode tip raises their temperature whereas the electrode itself is heated only passively. Once the electrode temperature is raised to the preset level the RF current is switched off and the repetition of this cycle maintains the desired temperature. During WC-RF application the specialized electrode used is actively cooled, by the continuous flow of water at ambient temperature. The active cooling prevents the electrode from acquiring the high surrounding tissue temperatures, and allows the continued flow of the RF current, with ensuing heating of larger tissue volume and a resultant larger thermal lesion [6]. Usual WC-RF lesion is comprised of few millimeters of cooled tissue immediately surrounding the electrode, encircled by spherical isotherms of rising temperature, followed by lower temperature isotherms [6]. Analogous to CT-RF lesion size, the size of WC-RF lesion depends on the probe size, electrode temperature, duration of RF current application, and the local tissue characteristics. While using 50°C isotherm as a criterion for lesion's edge, and if an 18-gauge electrode is used with its tip heated to 55°C to 60°C for 150 seconds, the resulting WC-RF lesion is roughly 8 to 10 mm in diameter [3, 4]. Even though a spherical area of tissue heating is expected [4], the local tissue features may influence the shape of the WC-RF lesion in vivo [6]. Active heat sinks, such as cerebrospinal fluid flow in thecal sac and blood flow in epidural venous plexus, and passive heat sinks, such as osseous and muscular spinal structures, may determine the eventual contour of the heated tissue [6].

CT-RF is currently the most frequently employed RF technique for the treatment of various pain syndromes. Electrode temperatures and lesion durations in use during CT-RF application are considerably discrepant in the published literature [7]. Although early investigational studies of CT-RF were suggestive of preferential small C-type nerve fiber damage [8], later studies showed indiscriminate damage to all nerve fiber types when tissue temperature was raised above 45°C [9]. Consequently, during CT-RF application injury to all neural fibers is expected and tissue temperature is therefore routinely raised to well above the neurodestructive point—80°C to 90°C [7]. Hence after CT-RF application functional impairment and exacerbation of pain by deafferentation phenomenon and local complications of nerve injury is expected. Because of these risks the use of CT-RF is conventionally limited to the treatment of facet syndrome. Pulsed RF is a newer modality which is used for a variety of pain syndromes. Although pulsed RF has been known to have relatively fewer adverse effects its efficacy is presently debatable [10].

The clinical use of WC-RF is at present limited to four recently published articles in the peer-reviewe journals [11–14]. In two of these studies [11, 12], WC-RF was applied for the treatment of sacroiliac joint dysfunction (SJD), and in two [13, 14] it pertained to the treatment of discogenic pain (DP). In a randomized controlled trial (RCT) of 28 patients with SJD [11], 14 patients in the treatment group received WC-RF of the S1, S2, and S3 lateral branches and CT-RF of L4 and L5 primary dorsal rami while 14 patients in the control group received the placebo treatment. Comparative analysis of the two study groups at one month showed statistically significant lower pain and disability scores in WC-RF treated patients. In the second study of SJD, retrospective analysis of 27 patients showed successful results with WC-RF application [12]. Of the two studies of WC-RF use in the treatment of DP, one is a prospective case series of 15 patients [13] and the other is a solitary patient case report [14]. In both these studies WC-RF was applied by placing two 17 G specialized electrodes in the postero lateral disc annulus, the electrode temperature was raised to 55°C over 11 minutes, and maintained at this level for further 4 minutes. Both the studies reported success of bipolar WC-RF in the treatment of DP [13, 14]. Accordingly, two distinct WC-RF techniques were identified, monopolar and bipolar WC-RF, used for the treatment of SJD and DP, respectively. 

The two cases reported here demonstrated that WC-RF may be employed when other neurolytic modalities have been ineffective. Additionally, we illustrated that the use of WC-RF may be extended to pain syndromes not commonly treated by common RF modalities. Although the success of WC-RF in the two cases we have reported is likely the result of larger thermal lesions created, other factors may have been implicated and should be further explored. Although the current evidence does not favor preferential disruption of small nociceptive fibers, the exiting evidence does not rule out such preferential nerve damage at temperatures between 40°C to 45°C [9]. Thermal lesioning at these temperatures may allow extirpation of pain sensation, without the risk of functional impairment and local nerve injury, as is the case with CT-RF. The ability of WC-RF to maintain temperatures of larger tissue volumes in a narrow desired range [3, 4, 6], may allow such neuromodulation of the nociceptive pathways. It is plausible that this mechanism of pain relief may have played a part in pain relief experienced by our second patient. The available thermal tissue mapping studies of WC-RF [3, 6, 14, 15] were conducted mainly to determine its safety and to confirm neurodestructive target tissue temperatures. No information is currently available on the electrode dimensions and current flow parameters requisite to achieve the target tissue temperatures in the narrow range of 40°C to 45°C. We recommend further exploration of the use of this novel RF modality.
